# A Germline Variant at 8q24 Contributes to the Serum p2PSA Level in a Chinese Prostate Biopsy Cohort

**DOI:** 10.3389/fonc.2021.753920

**Published:** 2021-10-19

**Authors:** Xiaoling Lin, Yishuo Wu, Fang Liu, Rong Na, Da Huang, Danfeng Xu, Jian Gong, Yao Zhu, Bo Dai, Dingwei Ye, Hongjie Yu, Haowen Jiang, Zujun Fang, Jie Zheng, Qiang Ding

**Affiliations:** ^1^ Department of Urology, Huashan Hospital, Fudan University, Shanghai, China; ^2^ Fudan Institute of Urology, Huashan Hospital, Fudan University, Shanghai, China; ^3^ Department of Urology, Ruijin Hospital, Shanghai Jiao Tong University School of Medicine, Shanghai, China; ^4^ Department of Urology, Shanghai Cancer Center, Fudan University, Shanghai, China; ^5^ State Key Laboratory of Genetic Engineering, School of Life Sciences, Fudan University, Shanghai, China

**Keywords:** genome-wide association study, p2PSA, polymorphism, prostate cancer, Chinese

## Abstract

**Introduction:**

The clinical performance of [–2]proPSA (p2PSA) and its derivatives in predicting the presence and aggressiveness of prostate cancer (PCa) has been well evaluated in prostate biopsy patients. However, no study has been performed to evaluate the common genetic determinants that affect serum level of p2PSA.

**Materials and Methods:**

Here, we performed a two-stage genome-wide association study (GWAS) on the p2PSA level in Chinese men who underwent a transperineal ultrasound-guided prostate biopsy at Huashan Hospital, Shanghai Cancer Center, and Ruijin Hospital in Shanghai, China. Germline variants significantly associated with the p2PSA level in the first stage (*n* = 886) were replicated in the second stage (*n* = 1,128). Multivariate linear regression was used to assess the independent contribution of confirmed single nucleotide polymorphisms (SNPs) and known covariates, such as age, to the level of p2PSA.

**Results:**

A novel non-synonymous SNP, rs72725879, in region 8q24.21 of the *PRNCR1* gene was significantly associated with the serum level of p2PSA in this two-stage GWAS (*p* = 2.28 × 10^−9^). Participants with homozygous “T” alleles at rs72725879 had higher p2PSA levels compared to allele “C” carriers. This variant was also nominally associated with PCa risk (*p*-combined = 3.44 × 10^−18^). The association with serum level of p2PSA was still significant after adjusting for PCa risk and age (*p* = 0.017).

**Conclusions:**

Our study shows that the genetic variants in the 8q24.21 region are associated with the serum level of p2PSA in a large-scale Chinese population. By taking inherited variations between individuals into account, the findings of these genetic variants may help improve the performance of p2PSA in predicting prostate cancer.

## Introduction

Prostate cancer (PCa) is one of the most common tumors in men and one of the leading causes of cancer-related death, conferring 1,111,700 new cases and 307,500 deaths annually worldwide (1). Although the incidence of PCa in Chinese is lower than that in Caucasian and African populations, it has been rising progressively in recent decades, along with increasing mortality (2).

Prostate-specific antigen (PSA) screening is the most widely used biomarker for the early detection and surveillance of PCa. However, PSA can also be affected by prostatitis, benign prostate hyperplasia, age, ethnicity, and genetic factors, which means it is organ-specific rather than cancer-specific. Therefore, its low specificity in clinical applications leads to quite a number of unnecessary biopsies and overdiagnosis of indolent cancers (3). In terms of genetic influence on the serum PSA level, it is estimated that 40% of the variations between individuals can be explained by inherited factors (4). In previous genome-wide association studies (GWAS), multiple inherited variants had been demonstrated to influence the serum levels of PSA in European and Asian populations (5–8).

Regarding the issues of unnecessary biopsies and overdiagnosis caused by PSA screening, new biomarkers with higher specificities and better abilities to discriminate PCa and aggressive PCa are needed. A relatively new biomarker [–2],proPSA (p2PSA), a predominant precursor form of PSA, was found elevated in almost all of the peripheral zone cancers, but was largely undetectable in the transition zone (9). In previous studies, p2PSA and its derivative prostate health index (phi) [(p2PSA/free PSA) × √tPSA] have been proven to have a better discriminating ability in predicting PCa in Caucasians (10–14). Later on, the clinical utilities of p2PSA and phi are also implicated in the Chinese (15–17). However, whether the serum p2PSA level is affected by genetic variance between different individuals remains unknown.

Discovery of novel genetic variants that influence the serum level of p2PSA may improve our understanding of the molecular mechanisms and clinical utility of p2PSA test. Therefore, to identify the genetic variants that influence the serum p2PSA level, we carried out a two-stage GWAS among Chinese men who underwent prostate biopsy.

## Material and Methods

### Study Cohorts and Design

Our study included two prostate biopsy cohorts from three medical centers, which were genotyped with the same genotyping array platform and denoted as stages 1 and 2.

Stage 1 consisted of 886 subjects from Huashan Hospital, Fudan University, and Shanghai Cancer Center, Fudan University, between 2010 and 2014. Stage 2 included 1,128 subjects from Huashan Hospital, Fudan University, and Ruijin Hospital, Shanghai Jiao Tong University School of Medicine, between 2015 and 2018. All the patients (*n* = 2,014) underwent initial prostate biopsies at the above-mentioned medical centers from 2010 to 2018.

The indications for prostate biopsy were the same across the three centers: 1) PSA >10.0 ng/ml; 2) PSA >4.0 ng/ml with a confirmation within 3 months; 3) PSA level ranging from 4.0 to 10.0 ng/ml, with suspicious %fPSA (free PSA divided by PSA) <0.16; and 4) abnormal findings from digital rectal examination (DRE), ultrasound, or magnetic resonance imaging (MRI) with any level of PSA.

Blood specimens were obtained before biopsies and serum samples were extracted. Serum total PSA (tPSA), free PSA (fPSA), and p2PSA were measured with a Beckman Coulter D×I 800 Immunoassay System (Beckman Coulter, Brea, CA, USA). All assays and quality control (QC) were performed according to the manufacturer’s instructions and standard QC protocols. Specifically, Access Hybritech Calibrators S0, S1–S6, i.e., blank and low to high concentrations, were run as internal known standards before each batch of the measurements. The measurements of the internal known control materials, Access Hybritech QC 1, 2, and 3, were below two standard deviations for each batch.

All epidemiological and clinical pieces of information were collected before biopsy. The patients would be excluded if pathologically diagnosed biopsy outcome was missing or if the tPSA, fPSA, and p2PSA were unable to be tested for bad quality. This study was approved by the institutional review board of each medical center, and written informed consent to participate in the present study was obtained from all participants.

### SNP Genotyping, Quality Control, Imputation, and GWAS Analysis

All DNA samples from stages 1 and 2 were extracted from blood samples and genotyped with the same genotyping array platform, Illumina Asian Screening Array (ASA) Beadchip, which included 659,184 single nucleotide polymorphisms (SNPs).

Genotyping QC was conducted together with data from stages 1 and 2. We used the following standard QC procedure to select qualified samples and SNPs for imputation analysis. Samples were excluded if they: i) had a genotyping call rate of <95%; ii) were duplicates or showed familial relationships [identity by state (IBS) >0.99]; and iii) had ambiguous gender. SNPs were removed if they had: i) a genotyping rate of <95% (*n* = 9,650); ii) a minor allele frequency of <0.01 (*n* = 152,901); and iii) a *p*-value <10^−3^ with the Hardy–Weinberg equilibrium test in patients with negative biopsy results (*n* = 2,721). After QC analysis, a total of 2,014 (886 in stage 1 and 1,128 in stage 2) samples with 493,912 genotyped SNPs were retained for imputation analysis.

Imputation was performed with the IMPUTE computer program (18) using the 1000 Genomes Project Han Chinese in Beijing (CHB) population as the reference. A total of 17,098,949 SNPs with imputation information score >0.90 were included in the analysis.

### Genome-Wide Association Analysis and Replication

GWAS analysis was conducted in stage 1, which included 886 samples with 17,098,949 SNPs. Using the same sample and SNP QC criteria above, 886 samples with 4,552,207 SNPs were left in the association analysis. PCAs were estimated using EIGENSTRAT. Beta values and *p*-values were estimated using quantitative linear regression for each SNP, adjusting for age and the first two PCAs.

We then performed a replication analysis using data from stage 2. As an independent set, stage 2 included 1,128 samples with 17,098,949 SNPs (including genotyped and imputed). The combined analysis of two-stage data was performed using linear regression, adjusting for age.

### Statistical Analysis

The associations between the serum p2PSA level and SNP genotypes were evaluated using a quantitative linear regression model assuming additive effects of the alleles (0, 1, and 2). In the regression models, log-transformed p2PSA levels were used as the dependent variable, each SNP as an independent variable, and age as a covariate. This analysis was performed by the PLINK V.1.90 software package (19). *P*-values less than 5 × 10^−8^ and 0.05 were regarded as significant levels in the GWAS and other analyses, respectively.

A principal component approach was used to evaluate population stratification in the first stage with the EIGENSTRAT software (20). The top two eigenvectors were adjusted as covariates in the quantitative linear regression analysis. Quantile–quantile (Q–Q) plots were performed using the R package (http://www.R-project.org). Linkage disequilibrium (LD)-based result clumping analysis was applied to test the independence of the respective SNPs in the 8q24.21 locus using PLINK (19). LocusZoom (21) and haploview (22) were used to create plots of genetic data.

## Results

A total of 886 subjects were recruited in the first stage and 1,128 in the second stage. The clinical characteristics of the cohorts in the two stages are described in **Table 1**. A total of 886 PCa cases were detected in two biopsy cohorts, with an overall positive biopsy rate of 42.9%. No significant difference in the clinical characteristics was observed between the two cohorts (**Table 1**).

**Table 1:** Characteristics of the participants in the two stages.

Characteristics	First stage (*N* = 886)				Second stage (*N* = 1,128)				*p*-value^c^
	All	Positive	Negative	*p*-value^b^	All	Positive	Negative	*p*-value^b^	
Biopsy result, *N* (%)	885	401 (45.31)	484 (54.69)		1124	462 (41.10)	662 (58.90)		0.68
p2PSA^a^, median (IQR), pg/ml	22.14 (49.56)	62.98 (312.52)	15.14 (13.97)	2.02 × 10^−68^	22.92 (40.02)	47.22 (181.97)	17.88 (16.73)	1.65 × 10^−52^	0.87
Age, mean ± SD, year	68.26 ± 9.64	70.87 ± 8.38	66.10 ± 10.05	1.17 × 10^−13^	67.73 ± 8.51	70.78 ± 7.92	65.60 ± 8.30	8.81 × 10^−24^	0.34

p2PSA [–2],proPSA; IQR, interquartile range; SD, standard deviation.

a p2PSA was measured in picograms per milliliter. Data were log-transformed for genome-wide association study (GWAS) analysis.

b P-values for the difference between positive and negative, Mann–Whitney U test for p2PSA, and t-test for age.

c P-values for the difference between two stages, chi-square test for the biopsy results, Mann–Whitney U test for p2PSA, and t-test for age.

**Figure 1:**
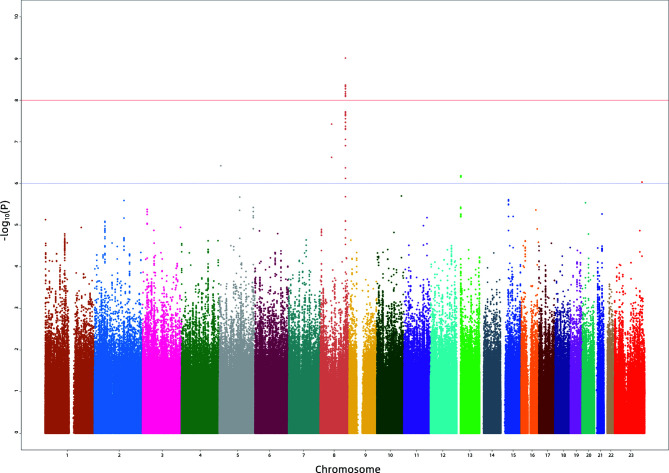
Manhattan plot for the genome-wide association study results for the levels of p2PSA in the Chinese population. The *X*-axis represents the chromosomal position and the *Y*-axis represents the –log10 *p*-value from linear regression. The *horizontal dashed line* shows the preset threshold of *p* = 1 × 10^−6^. The *horizontal solid line* indicates the preset threshold of *p* = 1 × 10^−8^.

In the first stage, 886 subjects were genotyped with the Illumina Asian Screening Array. After quality control, 4,552,207 SNPs and 877 individuals were eligible for GWAS analysis. We did not observe population structure in our cohort (**Supplementary Figure S1**). In addition, the Q-Q plots revealed an unadjusted inflation factor of 0.9999 (**Supplementary Figure S2**), indicating no evidence of systematic bias for the association of logp2PSA phenotype observed in the current study. The Manhattan plot of GWAS for the first stage is shown in **Figure 1**. We selected all signals associated with the logp2PSA level at *p* < 1 × 10^−5^ for replication analysis. A total of 148 SNPs were selected, and 86 of them reached a *p*-value of <5 × 10^−8^, which were mainly located in two regions, 8q21.3 and 8q24.21 (**Supplementary Table S1**).

In the second stage, 143 out of 148 SNPs remained qualified after QC in 1,128 subjects. Among the 143 candidate SNPs, rs72725879 at the 8q24 locus was confirmed to be significantly associated with the logp2PSA level at a *p*-value cutoff of 0.0003 (Bonferroni correction of 143 tests). We then combined the data of the two stages and found two SNPs that reached genome-wide significance (*p* = 2.28 × 10^−9^ for rs72725879 and *p* = 5.31 × 10^−9^ for rs13254738) (**Table 2**). Therefore, the 8q24.21 region was revealed to be significantly associated with p2PSA levels.

**Table 2:** Results of genome-wide association study (GWAS) for logp2PSA in the cohort.

SNP	CHR	Position^a^	Loci	Gene	Stage	A/a^b^	RAF^c^	Counts (aa/Aa/AA)^d^	Mean levels (pg/ml) (aa/Aa/AA)^d^	Beta (SE)	*p*-value^e^
rs72725879	8	128103969	8q24.21	*PRNCR1*	1	T/C	0.76	51/321/495	17.02/29.11/41.78	0.18 (0.037)	2.08 × 10^−6^
					2	T/C	0.76	70/393/636	22.23/27.10/38.90	0.12 (0.030)	6.24 × 10^−5^
					Combined	T/C	0.76	121/714/1131	19.86/27.99/40.18	0.14 (0.023)	2.28 × 10^−9^
rs13254738	8	128104343	8q24.21	*PRNCR1*	1	C/A	0.75	50/341/485	15.74/29.38/43.05	0.19 (0.037)	4.22 × 10^−7^
					2	C/A	0.76	73/403/648	27.16/25.94/38.82	0.10 (0.029)	3.90 × 10^−4^
					Combined	C/A	0.76	123/744/1133	21.78/27.48/40.64	0.13 (0.023)	5.31 × 10^−9^

a Chromosome position based on human genome build 37.

b A/a, Risk allele/reference allele.

c RAF indicates the frequency for risk allele A.

d aa, indicating homozygous with two reference alleles; Aa, heterozygous; AA, indicating homozygous with two risk alleles.

e P-value was based on multivariate linear regression analysis, adjusted for age and eigen.

In the current study, the strongest association effects were observed for two SNPs, rs72725879 and rs13254738, both of which were located in region 8q24.21 of the *PRNCR1* (PCa-associated non-coding RNA 1) gene. rs72725879-T and rs13254738-C showed a significant association with increasing serum levels of p2PSA. The two SNPs were correlated with each other (*R* ^2^ = 0.79). After adjusting the association results for rs72725879 as a covariate, rs13254738 became insignificant (*p* = 0.054). We then performed an additional univariate analysis for rs72725879 and found that it could explain 2.2% of the total genetic variance for the p2PSA levels.

Variants in the 8q24.21 region have previously been reported to be associated with a risk of PCa (23–30). Due to the potential confounding effects of the p2PSA level and PCa, we also evaluated whether the p2PSA-associated SNPs found in this study were associated with PCa. In the association analysis of the total study population combining the two cohorts, we confirmed the association of rs72725879 with PCa, with a combined odds ratio (OR) of 1.90 and a *p*-value of 3.44 × 10^−18^ for the T allele. After adjusting for PCa risk and age as covariates, rs72725879 was still associated with the p2PSA level (*p* = 0.017) (**Supplementary Table S2**).

We then grouped the subjects in the two stages into three groups based on their genotypes. The proportions of PCa cases detected in the CC, CT, and TT groups were 24.18%, 33.77%, and 50.76%, respectively. The T allele of rs72725879 showed a significant positive association with the increasing detection rate of PCa in the prostate biopsy cohort (*p*-_trend_ < 3.92 × 10^−21^) (**Supplementary Figure S3**).

**Figure 2:**
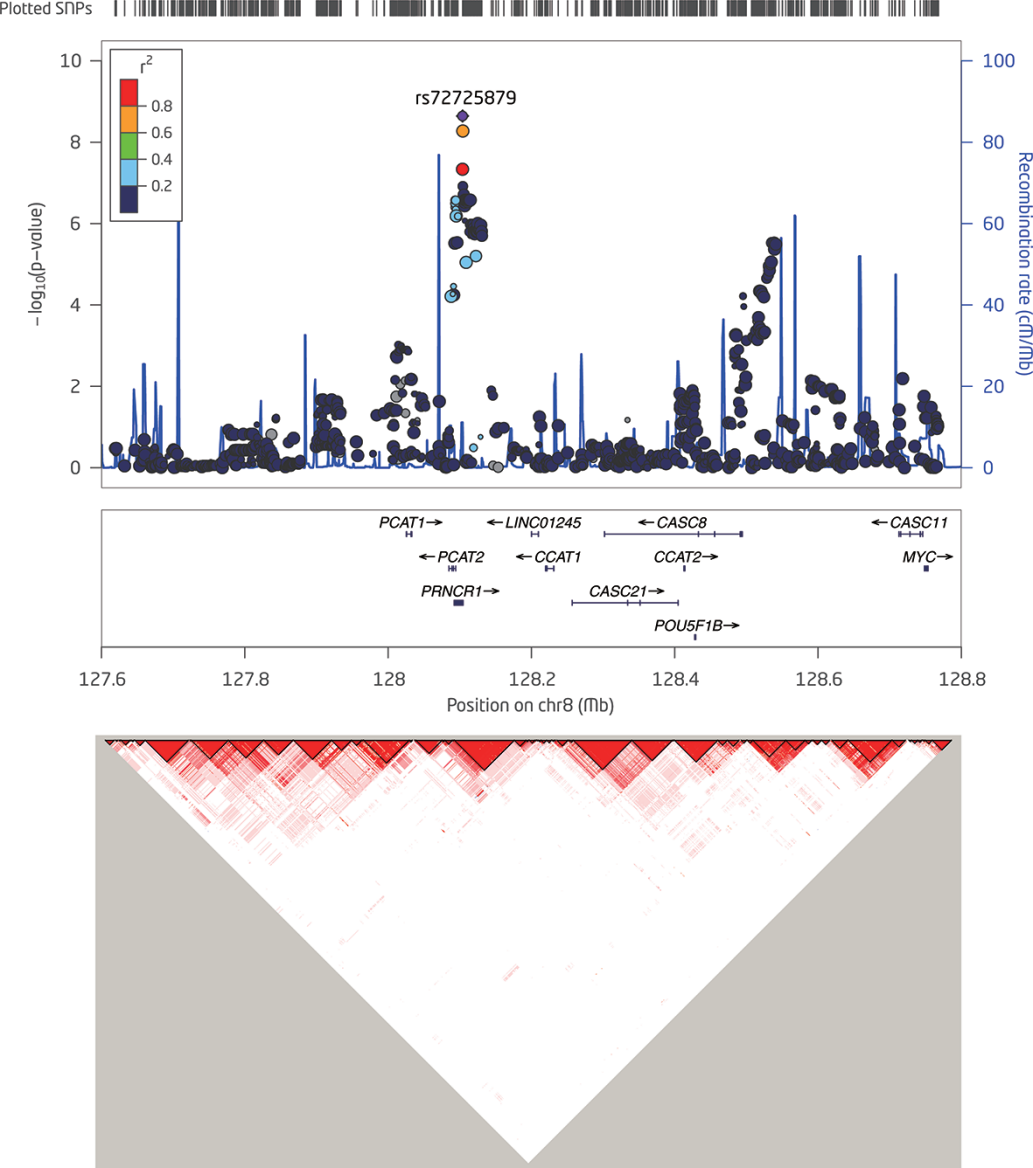
Detailed regional plots of −log10 *p*-values in the 8q24.21 region shown for logp2PSA. *Colors* indicate the linkage disequilibrium (LD) strength between rs7272589 and the other single nucleotide polymorphisms (SNPs) assessed. SNPs with *red circle* are reported to be associated with risk of prostate cancer (PCa) within the 8q24.21 region for the corresponding block. The *right Y*-axis shows the recombination rate from the 1000 Genomes Project data as reference. LD maps were based on *D*′ values using data from the two-stage samples.

Then, we specifically looked into 14 SNPs associated with a risk of PCa within the 8q24.21 region reported by previous GWAS and assessed their effects on the levels of p2PSA (**Table 3**). The Manhattan plot of 1,236 SNPs within this region is also shown in **Figure 2**. These 14 SNPs belonged to five LD blocks in this locus according to previously reported results (28). In the association analysis adjusted for age, we revealed that eight SNPs associated with PCa risk also contributed to the serum p2PSA level at a *p*-value cutoff of 0.0036 (after a Bonferroni correction of 14 tests), although it did not reach a GWAS significance. Among the eight SNPs, rs72725879 in block 2 was found to be in weak LD with rs13252298 (*r* ^2^ = 0.26) in the Chinese population (**Figure 2**).

**Table 3:** Association analysis between logp2PSA and prostate cancer (PCa) risk-associated variants reported by previous genome-wide association study (GWAS) within the region 8q24.21.

SNP	LD cluster^a^	CHR	BP^b^	Status	MA	*N*	Beta (SE)^c^	*p*-value^c^	PCa risk-associated SNPs identified by previous GWAS^d^			
									RA	OR	*p*-GWAS	PMID
rs12543663	Block 1	8	127,924,659	Genotyped	C	1,919	−0.056 (0.043)	0.20	C	1.12	2.60 × 10^−38^	29892016
rs10086908	Block 1	8	128,011,937	Genotyped	C	1,919	−0.083 (0.027)	1.82 × 10^−3^	T	1.13	1.80 × 10^−47^	29892016
rs1016343	Block 2	8	128,093,297	Genotyped	T	1,919	0.096 (0.02)	3.04 × 10^−6^	T	1.25	5.40 × 10^−21^	26034056
rs13252298	Block 2	8	128,095,156	Genotyped	G	1,919	−0.11 (0.022)	6.50 × 10^−7^	A	1.11	4.10 × 10^−10^	19767752
rs72725879	Block 2	8	128,103,969	Imputed	C	1,886	−0.14 (0.023)	2.28 × 10^−9^	NA	NA	NA	NA
rs6983561	Block 2	8	128,106,880	Genotyped	C	1,919	0.11 (0.022)	3.65 × 10^−7^	C	1.13	4.60 × 10^−19^	26034056
rs16901979	Block 2	8	128,124,916	Imputed	A	1,919	0.11 (0.022)	1.58 × 10^−6^	A	1.56	1.40 × 10^−105^	29892016
rs16902094	Block 2	8	128,320,346	Genotyped	G	1,919	0.0054 (0.023)	0.81	G	1.20	6.20 × 10^−15^	19767754
rs445114	Block 2	8	12,832,3181	Genotyped	C	1,919	0.0015 (0.020)	0.94	T	1.19	4.70 × 10^−10^	19767754
rs620861	Block 3	8	128,335,673	Genotyped	A	1,919	0.0093 (0.020)	0.65	G	1.15	4.90 × 10^−63^	29892016
rs16902104	Block 3	8	128,340,908	Imputed	T	1,827	0.0041 (0.023)	0.86	T	1.21	5.30 × 10^−10^	26034056
rs6983267	Block 4	8	128,413,305	Genotyped	G	1,919	0.045 (0.020)	0.026	G	1.22	2.80 × 10^−141^	29892016
rs1447295	Block 5	8	128,485,038	Genotyped	A	1,919	0.094 (0.027)	5.38 × 10^−4^	A	1.41	1.20 × 10^−179^	29892016
rs11986220	Block 5	8	128,531,689	Genotyped	A	1,896	0.12 (0.027)	1.13 × 10^−5^	A	1.56	2.30 × 10^−40^	26034056
rs7837688	Block 5	8	128,539,360	Genotyped	T	1,919	0.13 (0.027)	4.25 × 10^−6^	T	1.43	1.85 × 10^−14^	17401363

LD, linkage disequilibrium; CHR, chromosome; BP, base pair; MA, minor allele; RA, risk allele; OR, odds ratio; NA, not applicable.

a LD block information referring to the report by Olama et al. (28).

b Chromosome position based on human genome build 37.

c P-value, beta and standard error (SE) were based on multivariate linear regression analysis, adjusted for age.

d The RA, OR, and p-GWAS were from previously reported GWAS.

## Discussion

Previous studies have found more than 40 SNPs associated with the serum PSA level. These findings provided important information on the genetic variations in PSA among different individuals and could help improve personalized PSA screening, thereby reducing unnecessary biopsies. Besides PSA, a relatively new biomarker, p2PSA and its derivative phi, have become important biomarkers for PCa diagnosis, especially for men with a PSA level in the range 2.0–10.0 ng/ml. Yet, there is no study on the genetic variants influencing the serum p2PSA level. To our knowledge, this is the first GWAS on the serum p2PSA level in the Chinese population. In this two-stage GWAS in Chinese men who underwent prostate biopsy, we identified one single locus, 8q24.21, associated with the serum p2PSA level at genome-wide significance.

Multiple GWAS and fine-mapping studies had identified that common genetic variations in 8q24 influenced the inherited risk of PCa independently (24–30), while only one SNP (rs17464492) in 8q24 that influences the serum PSA level had been identified in non-Hispanic whites previously (7). In our study, rs72725879 (in region 2) appeared to be the leading SNP that affected the serum p2PSA level in the identified locus, 8q24.21, and it was located in the exon region of a non-coding RNA gene known as PCa-associated non-coding RNA 1 (*PRNCR1*). This SNP had previously been reported to be associated with PCa in men of African ancestry (30). According to data from The 1000 Genomes Project (1KGP), the risk allele frequency (RAF) of rs72725879 (T) was 0.76 in this study cohort, being higher than that in normal Asian (RAF = 0.66, ASN 1KGP), African (RAF = 0.33, AFR 1KGP), and European (RAF = 0.19, EUR 1KGP) populations. Previous GWAS had also identified that SNPs (rs1016343, rs13252298, rs13254738, and rs6983561) in *PRNCR1* were associated with PCa risk (23, 28, 29). In our two-stage combined analysis, SNP rs72725879 and the above three SNPs (rs1016343, rs13252298, and rs6983561) in *PRNCR1* were also found associated with PCa risk after adjustment for age (all *p* < 5.0 × 10^−8^). *PRNCR1*, also known as *PCAT8*, is a long non-coding RNA (lcRNA) that is upregulated in aggressive PCa. This lncRNA could bind to the androgen receptor and enhance the androgen receptor-mediated gene activation programs and proliferation in PCa cells (31). In the current study cohort, the T allele of rs72725879 was also observed to be associated with an increased risk of PCa detected in biopsy. Since a higher PCa risk can have an impact on the serum p2PSA level, it is also possible that the association between rs72725879 and the serum p2PSA level observed in the current study may also reflect some latent or undiagnosed disease.

Distinguishing whether SNPs are associated with p2PSA, PCa, or both is relatively complicated. The levels of p2PSA can be influenced by a number of factors (e.g., age, prostate infection, prostate inflammation, cancerous status, and urological manipulations). Nevertheless, we performed both the association analysis (with p2PSA) after adjusting for age and the association analysis with PCa to address part of these issues. Our results showed that the association between the genetic variant (rs72725879) and the p2PSA level was still significant after adjusting for PCa and age. This indicated that, although the association between PCa and serum p2PSA level was stronger, which is plausible because p2PSA is a diagnostic predictor for PCa, genetic variance also contributed to the baseline p2PSA level among different individuals. It has been reported that PSA-associated SNPs discovered in GWAS could be used to help normalize an individual’s PSA level, and incorporating these genetic factors into the application of PSA screening may increase the ability to classify individuals who should be biopsied (7). Here, we have proven that genetic variants also had an impact on p2PSA, so that personalizing the cutoff value for p2PSA by adjusting for genetic variants that were associated with the p2PSA levels in each individual might enhance the sensitivity and specificity of p2PSA in guiding biopsies.

There were several limitations in our study. Firstly, the study population was relatively small so that some signals might have been missed. Secondly, the overall p2PSA level in the current prostate biopsy cohort would be higher than that in a general population; thus, the findings from our study still need to be further validated in a larger general population. However, approximately 40% of the participants in our study were PCa cases, which enabled us to evaluate the associations between all p2PSA-associated SNPs and PCa.

## Conclusions

In the current study, we described the first GWAS in a Chinese prostate biopsy population and identified one single locus at 8q24.21 that was associated with the serum p2PSA level at genome-wide significance. By taking inherited variations between individuals into account, the findings of these genetic variants may help calculate personalized cutoff values for serum p2PSA for patients, thus improving the performance of p2PSA to predict PCa risk.

## Data Availability Statement

The original contributions presented in the study are included in the article/**Supplementary Materials**. Further inquiries can be directed to the corresponding authors.

## Ethics Statement

The studies involving human participants were reviewed and approved by the Institutional Review Board of Huashan Hospital, Fudan University; the Institutional Review Board of Shanghai Cancer Center, Fudan University; and the Institutional Review Board of Ruijin Hospital, Shanghai Jiao Tong University School of Medicine. The patients/participants provided written informed consent to participate in this study.

## Author Contributions

JZ, QD, and XL directed and designed the study. YW, FL, RN, DH, DX, JG, YZ, BD, DY, and HJ recruited the study subjects and managed the respective projects. XL and HY performed the bioinformatics and statistical analyses. XL and FL performed the genotyping and p2PSA testing. ZF and QD coordinated the project. All authors contributed to the article and approved the submitted version.

## Funding

This work was funded by grants from the Clinical Science and Technology Innovation Project of Shanghai Shen Kang Hospital Development Center to Qiang Ding (no. SHDC12015105), and Clinical Research Project of Shanghai Health Commission to YW (No.20214Y0511).

## Conflict of Interest

The authors declare that the research was conducted in the absence of any commercial or financial relationships that could be construed as a potential conflict of interest.

## Publisher’s Note

All claims expressed in this article are solely those of the authors and do not necessarily represent those of their affiliated organizations, or those of the publisher, the editors and the reviewers. Any product that may be evaluated in this article, or claim that may be made by its manufacturer, is not guaranteed or endorsed by the publisher.

## Supplementary Material

Supplementary Figure 1 The principal component analysis was conducted in 886 samples in the first stage and 1,978 from 1000 Genomes data by the first two principal components.

Supplementary Figure 2 The quantile-quantile (Q-Q) plot of the expected and observed *P* values using imputed SNPs data in stage 1.

Supplementary Figure 3 Plot for PCa detection rate by the genotypes of rs72725879.
