# Supplementary figures and images for: A Germline Variant at 8q24 Contributes to the Serum p2PSA Level in a Chinese Prostate Biopsy Cohort
Source: Front Oncol. 2021 Oct 19;11:753920. doi: 10.3389/fonc.2021.753920 (PMC8560794; doi:10.3389/fonc.2021.753920)

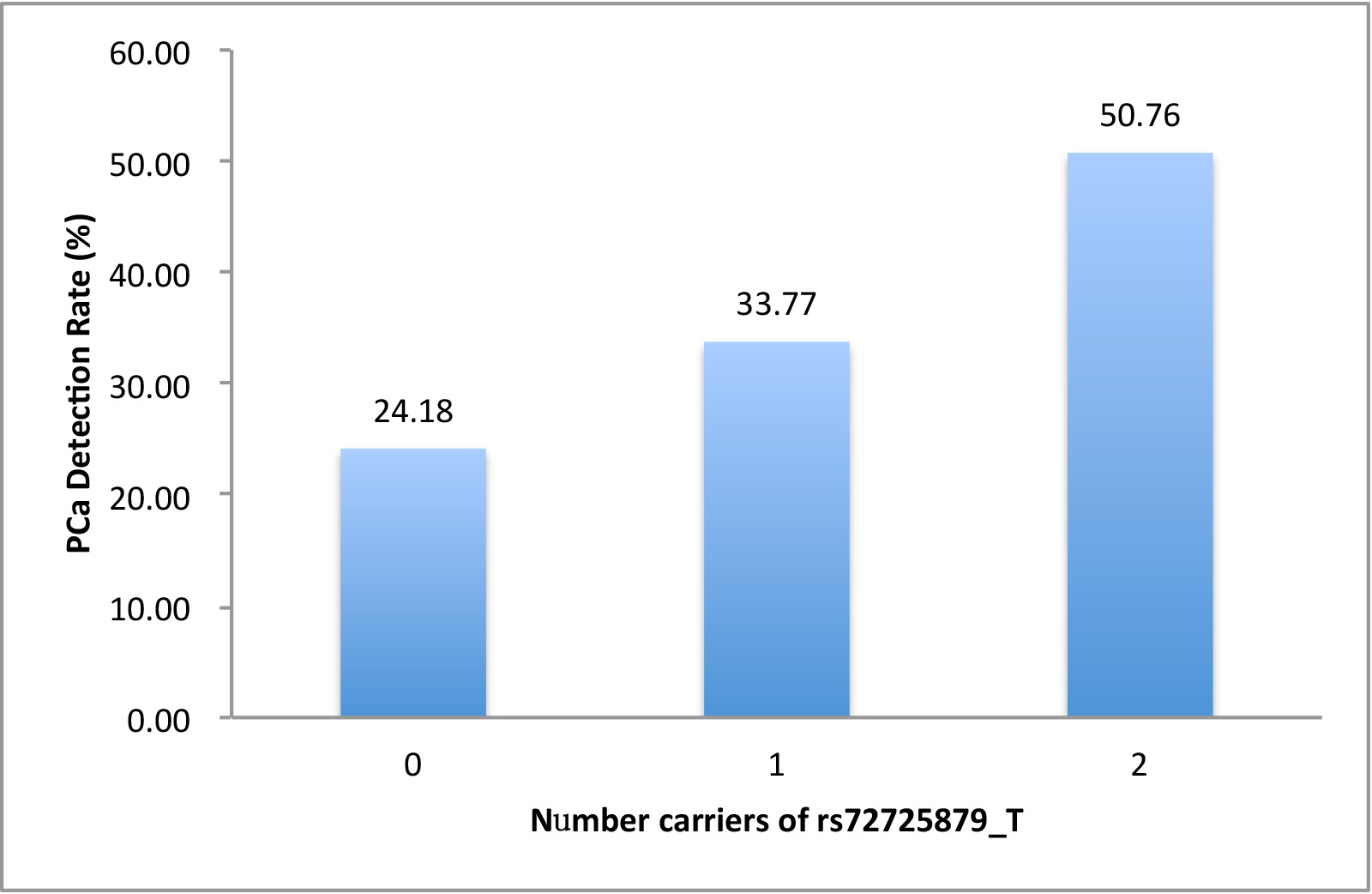

Supplement: Supplementary Figure 1 — The principal component analysis was conducted in 886 samples in the first stage and 1,978 from 1000 Genomes data by the first two principal components. [file Image_1.tif]

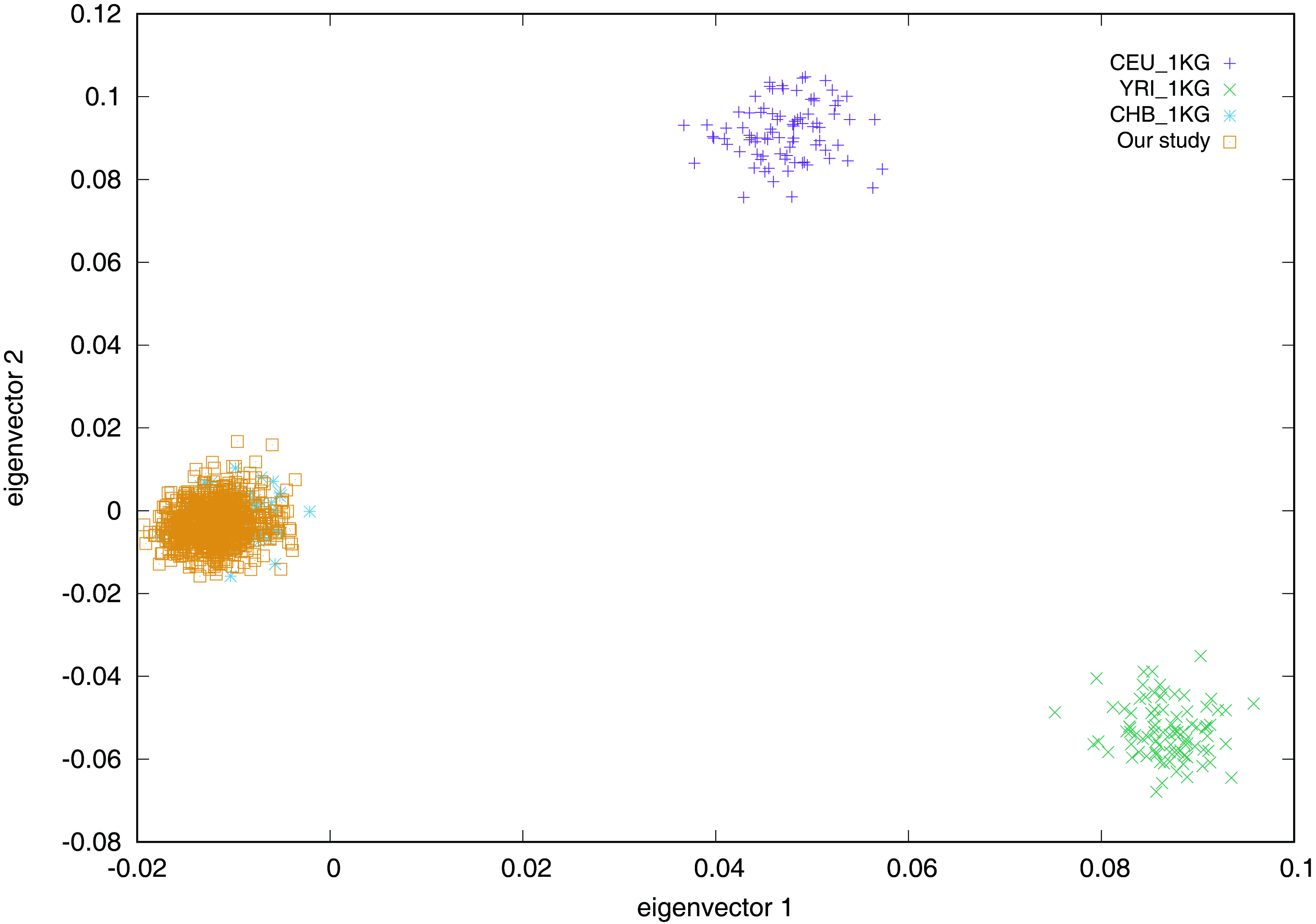

Supplement: Supplementary Figure 2 — The quantile-quantile (Q-Q) plot of the expected and observed P values using imputed SNPs data in stage 1. [file Image_2.tif]

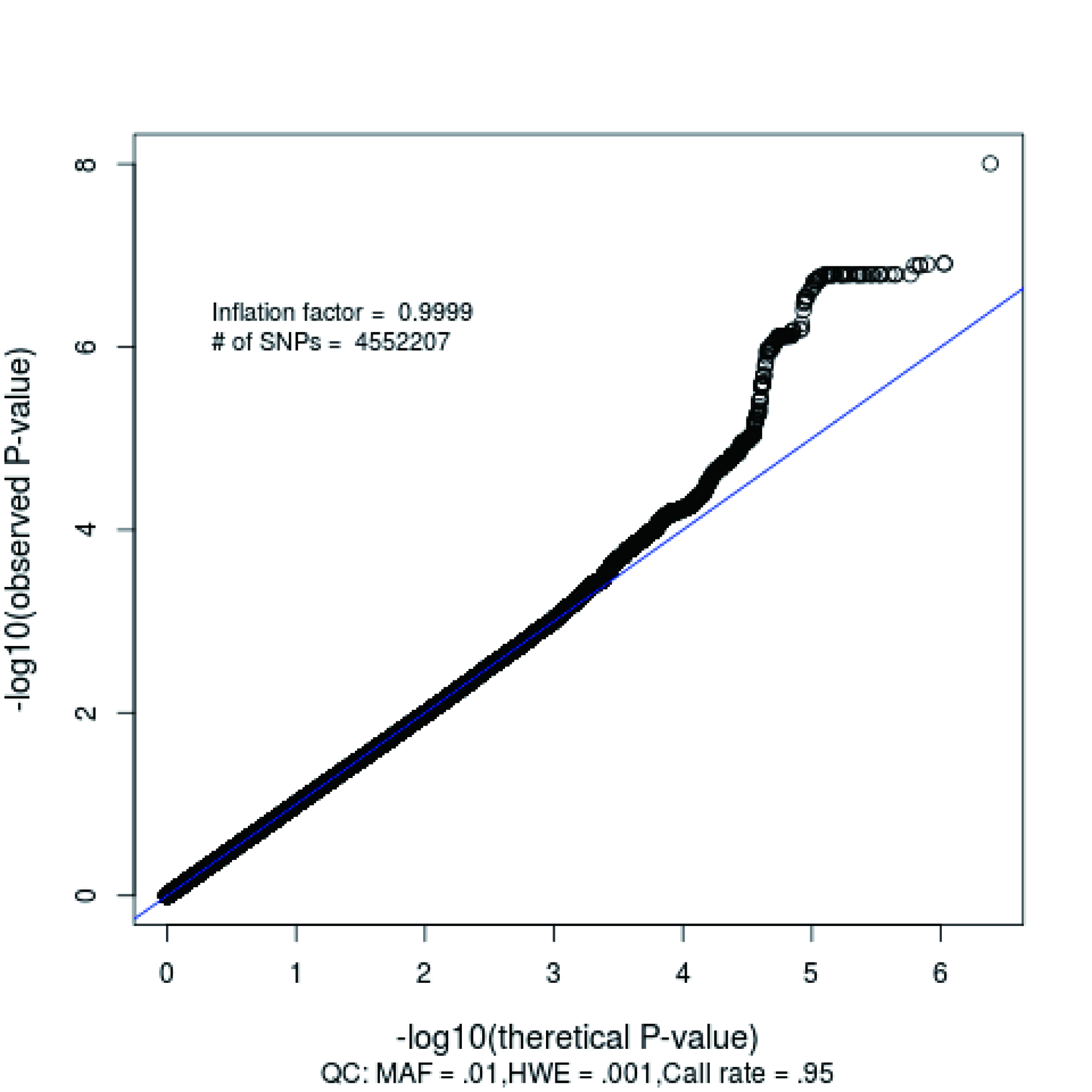

Supplement: Supplementary Figure 3 — Plot for PCa detection rate by the genotypes of rs72725879. [file Image_3.tif]
