# Supplementary material for: A Germline Variant at 8q24 Contributes to the Serum p2PSA Level in a Chinese Prostate Biopsy Cohort
Source: Front Oncol. 2021 Oct 19;11:753920. doi: 10.3389/fonc.2021.753920 (PMC8560794; doi:10.3389/fonc.2021.753920)
Supplement: Supplementary file 4 [file Table_1.doc]

| Supplementary Table 1: Association results of discovery stage for logp2PSA level. | | | | | | | |
| --- | --- | --- | --- | --- | --- | --- | --- |
| SNP | Position^a^ | Region | Status | MA^b^ | Beta | STAT | *P*- value^c^ |
| rs140440581 | 2529157 | 1p36.32 | Imputed | A | 0.3958 | 4.511 | 7.39 × 10^-6^ |
| rs1521938 | 53661243 | 2p16.2 | Imputed | C | 0.2112 | 4.491 | 8.13 × 10^-6^ |
| rs1494019 | 53661471 | 2p16.2 | Imputed | C | 0.2111 | 4.48 | 8.55 × 10^-6^ |
| rs56327237 | 150167113 | 2q23.2 | Imputed | A | 0.2654 | 4.53 | 6.80 × 10^-6^ |
| rs35757769 | 150194521 | 2q23.2 | Imputed | AT | 0.2889 | 4.737 | 2.57 × 10^-6^ |
| rs72619912 | 24168622 | 3p24.2 | Imputed | G | 0.2735 | 4.461 | 9.30 × 10^-6^ |
| rs3214790 | 24169631 | 3p24.2 | Imputed | GT | 0.2826 | 4.573 | 5.54 × 10^-6^ |
| rs72619913 | 24175261 | 3p24.2 | Imputed | A | 0.2881 | 4.632 | 4.21 × 10^-6^ |
| rs72619914 | 24176296 | 3p24.2 | Imputed | G | 0.2881 | 4.632 | 4.21 × 10^-6^ |
| rs72619915 | 24176687 | 3p24.2 | Imputed | T | 0.2881 | 4.632 | 4.21 × 10^-6^ |
| rs72619916 | 24179119 | 3p24.2 | Imputed | A | 0.2864 | 4.605 | 4.78 × 10^-6^ |
| rs58349927 | 24183586 | 3p24.2 | Imputed | C | 0.2786 | 4.457 | 9.45 × 10^-6^ |
| rs72619919 | 24184440 | 3p24.2 | Imputed | A | 0.2786 | 4.457 | 9.45 × 10^-6^ |
| rs72619920 | 24185519 | 3p24.2 | Imputed | G | 0.2778 | 4.465 | 9.13 × 10^-6^ |
| rs2924461 | 8012069 | 5p15.31 | Genotyped | G | 0.1681 | 5.122 | 3.76 × 10^-7^ |
| rs140963908 | 103168971 | 5q21.2 | Imputed | G | 0.3524 | 4.623 | 4.42 × 10^-6^ |
| rs80291192 | 103906989 | 5q21.2 | Imputed | G | 0.758 | 4.777 | 2.13 × 10^-6^ |
| rs522133 | 172431581 | 5q35.1 | Genotyped | T | 0.3155 | 4.656 | 3.77 × 10^-6^ |
| rs567969 | 172435016 | 5q35.1 | Imputed | A | 0.3175 | 4.607 | 4.73 × 10^-6^ |
| rs74637411 | 172436216 | 5q35.1 | Imputed | A | 0.3175 | 4.607 | 4.73 × 10^-6^ |
| rs10053713 | 172437641 | 5q35.1 | Genotyped | A | 0.305 | 4.534 | 6.63 × 10^-6^ |
| rs10062071 | 172438359 | 5q35.1 | Imputed | A | 0.3125 | 4.556 | 6.01 × 10^-6^ |
| rs113754584 | 57982359 | 8q21.3 | Imputed | T | 0.806 | 5.554 | 3.76 × 10^-8^ |
| 8_57983344 | 57983344 | 8q21.3 | Imputed | TTTTA | 0.7954 | 5.212 | 2.36 × 10^-7^ |
| rs6987760 | 128090311 | 8q24.21 | Imputed | T | -0.154 | -4.493 | 8.06 × 10^-6^ |
| rs34872094 | 128090701 | 8q24.21 | Imputed | C | -0.1541 | -4.497 | 7.93 × 10^-6^ |
| rs4581008 | 128090865 | 8q24.21 | Imputed | T | -0.1541 | -4.497 | 7.93 × 10^-6^ |
| rs12544839 | 128091382 | 8q24.21 | Imputed | A | -0.1539 | -4.496 | 7.97 × 10^-6^ |
| rs1031589 | 128092982 | 8q24.21 | Imputed | G | -0.1897 | -5.609 | 2.80 × 10^-8^ |
| rs1016343 | 128093297 | 8q24.21 | Genotyped | T | 0.169 | 5.334 | 1.24 × 10^-7^ |
| rs1031587 | 128093472 | 8q24.21 | Imputed | G | -0.1911 | -5.677 | 1.91 × 10^-8^ |
| rs1551510 | 128093478 | 8q24.21 | Imputed | C | -0.191 | -5.649 | 2.24 × 10^-8^ |
| rs4871008 | 128093541 | 8q24.21 | Imputed | T | -0.191 | -5.649 | 2.24 × 10^-8^ |
| 8_128093856 | 128093856 | 8q24.21 | Imputed | TAA | -0.1911 | -5.677 | 1.91 × 10^-8^ |
| rs6981122 | 128094460 | 8q24.21 | Imputed | A | -0.1884 | -5.512 | 4.78 × 10^-8^ |
| rs7001504 | 128094483 | 8q24.21 | Imputed | T | -0.1905 | -5.64 | 2.35 × 10^-8^ |
| rs13252298 | 128095156 | 8q24.21 | Genotyped | G | -0.1823 | -5.399 | 8.76 × 10^-8^ |
| rs7841060 | 128096477 | 8q24.21 | Imputed | G | 0.169 | 5.334 | 1.24 × 10^-7^ |
| rs6997559 | 128097578 | 8q24.21 | Imputed | C | -0.1885 | -5.503 | 5.04 × 10^-8^ |
| rs72725879 | 128103969 | 8q24.21 | Imputed | C | -0.1765 | -4.78 | 2.08 × 10^-6^ |
| rs13254738 | 128104343 | 8q24.21 | Genotyped | A | -0.1899 | -5.1 | 4.22 × 10^-7^ |
| rs7006390 | 128104367 | 8q24.21 | Imputed | T | 0.2014 | 5.832 | 7.88 × 10^-9^ |
| rs1073997 | 128105187 | 8q24.21 | Imputed | C | 0.2007 | 5.84 | 7.55 × 10^-9^ |
| rs7012442 | 128105731 | 8q24.21 | Imputed | G | 0.2009 | 5.855 | 6.91 × 10^-9^ |
| rs7016828 | 128106232 | 8q24.21 | Imputed | T | 0.1991 | 5.827 | 8.11 × 10^-9^ |
| rs7016830 | 128106235 | 8q24.21 | Imputed | G | 0.1991 | 5.827 | 8.11 × 10^-9^ |
| rs12682344 | 128106784 | 8q24.21 | Genotyped | G | 0.2006 | 5.871 | 6.28 × 10^-9^ |
| rs6983561 | 128106880 | 8q24.21 | Genotyped | C | 0.2016 | 5.899 | 5.35 × 10^-9^ |
| rs16901948 | 128107101 | 8q24.21 | Imputed | A | 0.2016 | 5.899 | 5.34 × 10^-9^ |
| rs16901949 | 128107153 | 8q24.21 | Imputed | C | 0.2016 | 5.899 | 5.34 × 10^-9^ |
| rs60681470 | 128107192 | 8q24.21 | Imputed | A | 0.2016 | 5.899 | 5.34 × 10^-9^ |
| rs16901950 | 128107243 | 8q24.21 | Imputed | A | 0.1937 | 5.536 | 4.23 × 10^-8^ |
| rs16901952 | 128107270 | 8q24.21 | Imputed | C | 0.2016 | 5.899 | 5.34 × 10^-9^ |
| rs58717512 | 128107381 | 8q24.21 | Imputed | A | 0.2026 | 5.933 | 4.37 × 10^-9^ |
| rs7005144 | 128107644 | 8q24.21 | Imputed | A | 0.2026 | 5.933 | 4.37 × 10^-9^ |
| rs145560166 | 128107647 | 8q24.21 | Imputed | AT | 0.2026 | 5.933 | 4.37 × 10^-9^ |
| rs59786353 | 128107682 | 8q24.21 | Imputed | C | 0.2026 | 5.933 | 4.37 × 10^-9^ |
| rs113924831 | 128108060 | 8q24.21 | Imputed | C | 0.2026 | 5.933 | 4.37 × 10^-9^ |
| rs16901953 | 128108229 | 8q24.21 | Imputed | C | 0.2026 | 5.933 | 4.37 × 10^-9^ |
| rs111723140 | 128108409 | 8q24.21 | Imputed | G | 0.2026 | 5.933 | 4.37 × 10^-9^ |
| rs200488072 | 128108414 | 8q24.21 | Imputed | T | 0.2026 | 5.933 | 4.37 × 10^-9^ |
| rs4871009 | 128108416 | 8q24.21 | Imputed | T | 0.1726 | 4.987 | 7.55 × 10^-7^ |
| rs61622455 | 128108516 | 8q24.21 | Imputed | C | 0.2026 | 5.933 | 4.37 × 10^-9^ |
| rs57435694 | 128108593 | 8q24.21 | Imputed | C | 0.2026 | 5.933 | 4.37 × 10^-9^ |
| rs7010450 | 128108679 | 8q24.21 | Imputed | G | 0.2026 | 5.933 | 4.37 × 10^-9^ |
| rs6990420 | 128108725 | 8q24.21 | Imputed | T | 0.2026 | 5.933 | 4.37 × 10^-9^ |
| rs73344342 | 128109356 | 8q24.21 | Imputed | A | 0.2026 | 5.933 | 4.37 × 10^-9^ |
| rs16901959 | 128109530 | 8q24.21 | Imputed | G | 0.2026 | 5.933 | 4.37 × 10^-9^ |
| rs7826388 | 128109776 | 8q24.21 | Imputed | T | 0.2026 | 5.933 | 4.37 × 10^-9^ |
| rs7830341 | 128109930 | 8q24.21 | Imputed | A | 0.2026 | 5.933 | 4.37 × 10^-9^ |
| rs16901966 | 128110252 | 8q24.21 | Genotyped | G | 0.2026 | 5.933 | 4.37 × 10^-9^ |
| rs16901967 | 128110277 | 8q24.21 | Imputed | G | 0.2026 | 5.933 | 4.37 × 10^-9^ |
| rs7001069 | 128110646 | 8q24.21 | Imputed | G | 0.2023 | 5.92 | 4.74 × 10^-9^ |
| rs7006076 | 128111238 | 8q24.21 | Imputed | A | 0.2023 | 5.92 | 4.74 × 10^-9^ |
| rs7005862 | 128111342 | 8q24.21 | Imputed | G | 0.2023 | 5.92 | 4.74 × 10^-9^ |
| rs7006251 | 128111343 | 8q24.21 | Imputed | A | 0.2023 | 5.92 | 4.74 × 10^-9^ |
| rs6988257 | 128111456 | 8q24.21 | Imputed | C | 0.2026 | 5.933 | 4.37 × 10^-9^ |
| rs73348236 | 128111470 | 8q24.21 | Imputed | T | 0.2026 | 5.933 | 4.37 × 10^-9^ |
| rs57010632 | 128111717 | 8q24.21 | Imputed | G | 0.2026 | 5.933 | 4.37 × 10^-9^ |
| rs16901969 | 128112097 | 8q24.21 | Imputed | C | 0.2026 | 5.933 | 4.37 × 10^-9^ |
| rs73348241 | 128112179 | 8q24.21 | Imputed | A | 0.2026 | 5.933 | 4.37 × 10^-9^ |
| rs73348243 | 128112193 | 8q24.21 | Imputed | A | 0.2026 | 5.933 | 4.37 × 10^-9^ |
| rs59294648 | 128112229 | 8q24.21 | Imputed | TAAG | 0.2026 | 5.933 | 4.37 × 10^-9^ |
| rs61265543 | 128112446 | 8q24.21 | Imputed | T | 0.2026 | 5.933 | 4.37 × 10^-9^ |
| rs16901970 | 128112715 | 8q24.21 | Imputed | G | 0.2026 | 5.933 | 4.37 × 10^-9^ |
| rs10453084 | 128112779 | 8q24.21 | Imputed | A | 0.2026 | 5.933 | 4.37 × 10^-9^ |
| rs6987723 | 128112859 | 8q24.21 | Imputed | A | 0.2026 | 5.933 | 4.37 × 10^-9^ |
| rs6987640 | 128113028 | 8q24.21 | Imputed | T | 0.2026 | 5.933 | 4.37 × 10^-9^ |
| rs56005245 | 128113426 | 8q24.21 | Imputed | T | 0.1992 | 6.186 | 9.78 × 10^-10^ |
| rs74305597 | 128113464 | 8q24.21 | Imputed | T | 0.2026 | 5.933 | 4.37 × 10^-9^ |
| rs7824451 | 128114461 | 8q24.21 | Imputed | G | 0.2026 | 5.933 | 4.37 × 10^-9^ |
| rs7824785 | 128114710 | 8q24.21 | Imputed | T | 0.2026 | 5.933 | 4.37 × 10^-9^ |
| rs73348257 | 128115259 | 8q24.21 | Imputed | A | 0.1909 | 5.572 | 3.41 × 10^-8^ |
| rs6470498 | 128115720 | 8q24.21 | Imputed | G | 0.1909 | 5.572 | 3.41 × 10^-8^ |
| rs6999725 | 128115752 | 8q24.21 | Imputed | T | 0.1909 | 5.572 | 3.41 × 10^-8^ |
| rs57462547 | 128117863 | 8q24.21 | Imputed | T | 0.1909 | 5.572 | 3.41 × 10^-8^ |
| rs7844219 | 128118815 | 8q24.21 | Imputed | G | 0.1909 | 5.572 | 3.41 × 10^-8^ |
| rs6982324 | 128119056 | 8q24.21 | Imputed | C | 0.1909 | 5.572 | 3.41 × 10^-8^ |
| rs140266678 | 128119887 | 8q24.21 | Imputed | AAG | 0.1944 | 5.644 | 2.29 × 10^-8^ |
| rs187359375 | 128120151 | 8q24.21 | Imputed | T | 0.1955 | 5.671 | 1.97 × 10^-8^ |
| rs34425474 | 128120234 | 8q24.21 | Imputed | C | 0.1944 | 5.644 | 2.29 × 10^-8^ |
| rs368538194 | 128120421 | 8q24.21 | Imputed | C | 0.1955 | 5.671 | 1.97 × 10^-8^ |
| rs201821183 | 128120465 | 8q24.21 | Imputed | T | 0.1952 | 5.666 | 2.02 × 10^-8^ |
| rs371764913 | 128120718 | 8q24.21 | Imputed | T | 0.1943 | 5.639 | 2.35 × 10^-8^ |
| rs201401963 | 128120772 | 8q24.21 | Imputed | G | 0.1943 | 5.639 | 2.35 × 10^-8^ |
| rs4341134 | 128121288 | 8q24.21 | Imputed | C | 0.1943 | 5.639 | 2.35 × 10^-8^ |
| rs6470499 | 128123394 | 8q24.21 | Imputed | C | 0.1944 | 5.644 | 2.29 × 10^-8^ |
| rs7842760 | 128123470 | 8q24.21 | Imputed | A | 0.1944 | 5.644 | 2.29 × 10^-8^ |
| rs148913765 | 128123830 | 8q24.21 | Imputed | CA | 0.1944 | 5.644 | 2.29 × 10^-8^ |
| rs1551512 | 128124126 | 8q24.21 | Imputed | G | 0.1944 | 5.644 | 2.29 × 10^-8^ |
| rs16901979 | 128124916 | 8q24.21 | Imputed | A | 0.1944 | 5.644 | 2.29 × 10^-8^ |
| rs10505483 | 128125195 | 8q24.21 | Genotyped | T | 0.1944 | 5.644 | 2.29 × 10^-8^ |
| rs7817677 | 128125504 | 8q24.21 | Imputed | G | 0.1944 | 5.644 | 2.29 × 10^-8^ |
| rs73350138 | 128126798 | 8q24.21 | Imputed | G | 0.1944 | 5.644 | 2.29 × 10^-8^ |
| rs72154702 | 128127819 | 8q24.21 | Imputed | AC | 0.1944 | 5.644 | 2.29 × 10^-8^ |
| rs12678505 | 128128506 | 8q24.21 | Imputed | T | 0.1944 | 5.644 | 2.29 × 10^-8^ |
| rs6989838 | 128129372 | 8q24.21 | Imputed | C | 0.1944 | 5.644 | 2.29 × 10^-8^ |
| rs200548274 | 128129985 | 8q24.21 | Imputed | GAATC | 0.1944 | 5.644 | 2.29 × 10^-8^ |
| rs7013255 | 128130487 | 8q24.21 | Imputed | G | 0.1944 | 5.644 | 2.29 × 10^-8^ |
| rs16901984 | 128130961 | 8q24.21 | Imputed | C | 0.1956 | 5.654 | 2.16 × 10^-8^ |
| rs11200425 | 123895174 | 10q26.13 | Imputed | T | 0.371 | 4.788 | 2.00 × 10^-6^ |
| rs11216572 | 117701836 | 11q23.3 | Imputed | C | -0.1623 | -4.535 | 6.64 × 10^-6^ |
| 13_19696338 | 19696338 | 13q12.11 | Imputed | A | 0.6162 | 4.644 | 3.98 × 10^-6^ |
| rs185256623 | 20023865 | 13q12.11 | Imputed | C | 0.5866 | 4.545 | 6.33 × 10^-6^ |
| 13_20042364 | 20042364 | 13q12.11 | Imputed | G | 0.5881 | 4.555 | 6.04 × 10^-6^ |
| rs147478483 | 20141161 | 13q12.11 | Imputed | A | 0.589 | 4.57 | 5.62 × 10^-6^ |
| rs117465141 | 20198720 | 13q12.11 | Imputed | A | 0.5899 | 4.577 | 5.45 × 10^-6^ |
| rs114727280 | 20292578 | 13q12.11 | Imputed | T | 0.6685 | 5 | 7.02 × 10^-7^ |
| rs3858741 | 20292600 | 13q12.11 | Imputed | G | 0.6685 | 5 | 7.02 × 10^-7^ |
| 13_20350933 | 20350933 | 13q12.11 | Imputed | G | 0.6697 | 5.014 | 6.55 × 10^-7^ |
| rs118045216 | 20396716 | 13q12.11 | Imputed | T | 0.6697 | 5.014 | 6.55 × 10^-7^ |
| rs147579240 | 20477197 | 13q12.11 | Imputed | A | 0.6692 | 5.007 | 6.77 × 10^-7^ |
| rs34356257 | 38989605 | 15q14 | Imputed | C | 0.4493 | 4.732 | 2.62 × 10^-6^ |
| rs34141940 | 38989657 | 15q14 | Imputed | A | 0.4493 | 4.732 | 2.62 × 10^-6^ |
| rs34281605 | 38989674 | 15q14 | Imputed | A | 0.4493 | 4.732 | 2.62 × 10^-6^ |
| rs35024396 | 38989770 | 15q14 | Imputed | A | 0.4493 | 4.732 | 2.62 × 10^-6^ |
| rs2172835 | 38990171 | 15q14 | Imputed | T | 0.4493 | 4.732 | 2.62 × 10^-6^ |
| rs12916379 | 38991520 | 15q14 | Imputed | G | 0.4493 | 4.732 | 2.62 × 10^-6^ |
| rs2132157 | 38992547 | 15q14 | Imputed | G | 0.4493 | 4.732 | 2.62 × 10^-6^ |
| rs12901215 | 38992893 | 15q14 | Imputed | A | 0.4459 | 4.745 | 2.46 × 10^-6^ |
| rs7183893 | 38996981 | 15q14 | Imputed | C | 0.4197 | 4.548 | 6.23 × 10^-6^ |
| rs36094159 | 38998395 | 15q14 | Imputed | A | 0.5153 | 4.694 | 3.15 × 10^-6^ |
| rs35795311 | 38998669 | 15q14 | Imputed | G | 0.5148 | 4.687 | 3.26 × 10^-6^ |
| rs17304240 | 62689308 | 15q22.2 | Imputed | T | 0.2485 | 4.549 | 6.21 × 10^-6^ |
| rs79946007 | 75138273 | 16q23.1 | Imputed | A | 0.6847 | 4.627 | 4.33 × 10^-6^ |
| rs6045053 | 17869300 | 20p12.1 | Genotyped | A | -0.1819 | -4.71 | 2.91 × 10^-6^ |
| rs2835694 | 38671630 | 21q22.13 | Imputed | G | 0.167 | 4.578 | 5.42 × 10^-6^ |
| rs4322130 | 141199644 | Xq27.2 | Genotyped | G | 1.065 | 4.944 | 9.29 × 10^-7^ |

^a^ Chromosome position based on human genome build 37.

^b^MA indicates the minor allele.

^c^*P*-value was based on multivariate linear regression analysis, adjusted for age and eigen.
