# Supplementary material for: A Germline Variant at 8q24 Contributes to the Serum p2PSA Level in a Chinese Prostate Biopsy Cohort
Source: Front Oncol. 2021 Oct 19;11:753920. doi: 10.3389/fonc.2021.753920 (PMC8560794; doi:10.3389/fonc.2021.753920)
Supplement: Supplementary file 5 [file Table_2.docx]

Supplementary Table 2 Association results between rs72725879 and Logp2PSA levels adjusted for Age and PCa.

| CHR | SNP | BP^a^ | MA^b^ | TEST | NMISS | BETA | SE | L95 | U95 | *P*-value^c^ |
| --- | --- | --- | --- | --- | --- | --- | --- | --- | --- | --- |
| 8 | rs72725879 | 128103969 | C | ADD | 1882 | -0.050 | 0.021 | -0.090 | -0.0088 | 0.017 |
| 8 | rs72725879 | 128103969 | C | Age | 1882 | 0.0067 | 0.0014 | 0.0039 | 0.0095 | 3.58 × 10^-6^ |
| 8 | rs72725879 | 128103969 | C | PCa | 1882 | 0.62 | 0.027 | 0.56 | 0.67 | 2.86 × 10^-103^ |

^a^ Chromosome position based on human genome build 37.

^b^MA indicates the minor allele.

^c^*P*-value was based on multivariate linear regression analysis, adjusted for age and PCa risk.
